# Novel ATP‐competitive Akt inhibitor afuresertib suppresses the proliferation of malignant pleural mesothelioma cells

**DOI:** 10.1002/cam4.1179

**Published:** 2017-09-27

**Authors:** Masayuki Yamaji, Akinobu Ota, Md Wahiduzzaman, Sivasundaram Karnan, Toshinori Hyodo, Hiroyuki Konishi, Shinobu Tsuzuki, Yoshitaka Hosokawa, Masayuki Haniuda

**Affiliations:** ^1^ Division of Chest Surgery Department of Surgery Aichi Medical University School of Medicine Nagakute Aichi 480‐1195 Japan; ^2^ Department of Biochemistry Aichi Medical University School of Medicine Nagakute Aichi 480‐1195 Japan

**Keywords:** Afuresertib, Akt inhibitor, cancer therapy, Mesothelioma

## Abstract

Malignant pleural mesothelioma (MPM), an asbestos‐related occupational disease, is an aggressive and incurable tumor of the thoracic cavity. Despite recent advances in MPM treatment, overall survival of patients with MPM is very low. Recent studies have implicated that PI3K/Akt signaling is involved in MPM cell survival and development. To investigate the effects of Akt inhibitors on MPM cell survival, we examined the effects of nine selective Akt inhibitors, namely, afuresertib, Akti‐1/2, AZD5363, GSK690693, ipatasertib, MK‐2206, perifosine, PHT‐427, and TIC10, on six MPM cell lines, namely, ACC‐MESO‐4, Y‐MESO‐8A, MSTO‐211H, NCI‐H28, NCI‐H290, and NCI‐H2052, and a normal mesothelial cell line MeT‐5A. Comparison of IC_50_ values of the Akt inhibitors showed that afuresertib, an ATP‐competitive specific Akt inhibitor, exerted tumor‐specific effects on MPM cells. Afuresertib significantly increased caspase‐3 and caspase‐7 activities and apoptotic cell number among ACC‐MESO‐4 and MSTO‐211H cells. Moreover, afuresertib strongly arrested the cell cycle in the G_1_ phase. Western blotting analysis showed that afuresertib increased the expression of p21^WAF^ ^1/^ ^CIP^ ^1^ and decreased the phosphorylation of Akt substrates, including GSK‐3*β* and FOXO family proteins. These results suggest that afuresertib‐induced p21 expression promotes G_1_ phase arrest by inducing FOXO activity. Furthermore, afuresertib significantly enhanced cisplatin‐induced cytotoxicity. Interestingly, results of gene set enrichment analysis showed that afuresertib modulated the expression *E2F1* and *MYC*, which are associated with fibroblast core serum response. Together, these results suggest that afuresertib is a useful anticancer drug for treating patients with MPM.

## Introduction

Malignant pleural mesothelioma (MPM) is an incurable thoracic malignancy, with a gradually increasing incidence worldwide because of asbestos exposure 1, 2. Despite the availability of different options for treating patients with MPM, including surgery, radiotherapy, and chemotherapy, median survival of these patients ranges between 6 and 9 months after diagnosis, indicating poor prognosis 3. Recent studies implicated that several oncogenic somatic events are involved in MPM development 4, 5. Whole‐exon sequencing identified frequent genetic alterations in *BAP1*,* NF2*,* CDKN2A*, and *CUL1* in patients with MPM 4. Activation of Hippo‐Yes‐associated protein/transcriptional coactivator with PDZ‐binding motif (YAP/TAZ) signaling plays an important role in MPM cell proliferation 5. Although several molecules associated with cancer development have been identified, an efficient molecular targeting therapy for treating patients with MPM remains to be developed. Therefore, effective clinical approaches are required for treating MPM.

Akt (protein kinase B) is a master regulator of cell survival in response to growth factors 6, 7. In human cancers, Akt plays a pivotal role in cell growth, apoptosis inhibition, protein synthesis, and glucose and fatty acid metabolism by phosphorylating its substrates, including CDK2, FOXO, GSK‐3beta, S6 kinase, and mTOR 8. These processes are frequently activated in various solid and hematologic malignancies. In addition, Akt phosphorylates YAP/TAZ, which induces mesothelioma cell proliferation by upregulating the expression of cell cycle‐promoting genes 5 and suppressing the expression of proapoptotic *BAX* 9. Akt activation induces resistance to trastuzumab 10, 11, 12, hormonal treatment for breast 13, 14, 15 and prostate cancers 16, 17, 18, and chemotherapy 19, 20, 21. Thus, accumulating evidence implicate that Akt signaling is one of the most promising targets for cancer therapy.

To date, several compounds have been shown to inhibit the molecular action of Akt and tumor cell growth 8. Recent studies clinically evaluated perifosine and afuresertib for treating patients with relapsed myeloma 22, 23. In addition, GDC‐0068 is currently in preclinical development for treating triple‐negative breast cancer 24. Because inhibition of Akt signaling is an attractive target for cancer therapy, it is interesting to examine the effects of Akt inhibitors on MPM cell growth.

In this study, we found that afuresertib preferentially inhibited the proliferation of, significantly induced the apoptosis of, and enhanced cisplatin‐induced cytotoxicity against MPM cells. Moreover, we determined molecular mechanism underlying the antitumor effects of afuresertib.

## Materials and Methods

### Reagents

RPMI1640 medium and penicillin‐streptomycin were purchased from Wako Pure Chemical Industries, Ltd (Osaka, Japan). FBS was obtained from Nichirei Biosciences Inc. (Tokyo, Japan). MTT was obtained from Sigma‐Aldrich (St. Louis, MO). Akt inhibitors afuresertib, Akti‐1/2, AZD5363, GSK690693, ipatasertib (GDC‐0068), MK‐2206, perifosine, PHT‐427, and TIC10 and PDPK1 inhibitor OSU‐03012 were obtained from Selleck Chemicals (Houston, TX). Propidium iodide (PI) was obtained from Merck Millipore (Billerica, MA). Annexin V (AxV)–FITC was obtained from MBL (Nagoya, Japan). All antibodies used in this study were purchased from Cell Signaling Technology Inc. (Beverly, MA).

### Cell culture

Six MPM cell lines, namely, ACC‐MESO‐4, MSTO‐211H, NCI‐H2052, NCI‐H28, NCI‐H290, and Y‐MESO‐8A, and a normal mesothelial cell line MeT‐5A were kindly provided by Dr. Y. Sekido, Division of Molecular Oncology, Aichi Cancer Center Research Institute. The cell lines were maintained in RPMI1640 medium supplemented with 10% heat‐inactivated FBS and penicillin–streptomycin at 37°C in a humidified atmosphere of 5% CO_2_. The cells were detached from 90‐mm dishes using trypsin and were seeded in 96‐, 24‐, 12‐, or 6‐well plates for experimental purposes.

### Cell viability assay

MPM cells were seeded in 96‐well plates (cell density, 2.5 × 10^3^ cells/well) and were incubated for 24 h at 37°C. Next, the cells were incubated in a medium containing indicated concentrations of Akt inhibitors for 72 h. Next, MTT solution was added to each well, and the cells were incubated for 4 h. Finally, the cells were incubated overnight with lysis buffer (10% SDS in 0.01 mol/L hydrogen chloride). Absorbance was measured at 550 nm using SpectraMAX M5 spectrophotometer (Molecular Devices, Sunnyvale, CA).

### Apoptosis assay

Apoptosis was evaluated by performing AxV–FITC/PI double staining‐based FACS analysis, as described previously 25. Briefly, ACC‐MESO‐4 and MSTO‐211H cells were seeded in six‐well plates (cell density, 1 × 10^5^ cells/well) and were incubated for 24 h at 37°C. Next, the cells were incubated with indicated concentrations of afuresertib, followed by incubation with AxV–FITC and PI (10 *μ*g/mL) for 15 min at room temperature. Fluorescence intensities were determined by performing FACS with FACSCantoII (BD, Franklin Lakes, NJ).

### Cell cycle analysis

Cell cycle was evaluated by performing PI‐staining‐based FACS analysis, as described previously 26. ACC‐MESO‐4 and MSTO‐211H cells were seeded in a six‐well culture plate (cell density, 1 × 10^5^ cells/well) and were incubated for 24 h. Next, the cells were incubated with the indicated concentrations of afuresertib for 24 h. For FACS analysis, the cells were detached using trypsin after 24 h of serum treatment and were fixed overnight in ice‐cold 70% ethanol. After fixation, the cells were treated with RNase A (100 *μ*g/mL) and stained with PI (10 *μ*g/mL). The percentages of cells in the sub‐G_1_, G_1_, S, and G_2_‐M phases of the cell cycle were measured using FlowJo software (Tree Star Inc.; Ashland, OR USA).

### Cell confluence proliferation assay

Cell confluence proliferation assay was performed using Incucyte ZOOM^®^ Live‐Cell Imaging System (Essen Bioscience, Ann Arbor, MI), according to the manufacture's instruction. Briefly, ACC‐MESO‐4 and MSTO‐211H cells were seeded in 12‐well plates (cell density, 1 × 10^4^ cells/well) and were incubated for 24 h at 37°C. Then, the cells were incubated with 0, 2, 5, or 10 *μ*mol/L afuresertib. During treatment, cell growth was monitored by recording phase images using the IncuCyte ZOOM^®^ Live‐Cell Imaging System (magnification, ×100).

### Scratching assay

ACC‐MESO‐4 and MSTO‐211H cells were seeded in 24‐well plates (cell density, 1 × 10^5^ cells/well) and were incubated for 24 h at 37°C. After the cells reached 80–90% confluence, they were gently scratched with a new 1 mL pipette tip across the center of the well. After scratching, cell culture medium was replaced with a fresh medium containing the indicated concentration of afuresertib. Visual validation of the treatment was performed using the IncuCyte^™^ ZOOM Living‐Cell Imaging System (Essen BioScience, Inc., Ann Arbor, MI).

### Quantitative RT‐PCR analysis

For performing quantitative RT‐PCR (qRT‐PCR), ACC‐MESO‐4 and MSTO‐211H cells were seeded in six‐well plates (cell density, 1 × 10^5^ cells/well) and were incubated for 24 h at 37°C. Next, the cells were incubated with the indicated concentrations of afuresertib. After incubation for 24 h, total RNA was extracted from the cells using NucleoSpin^®^ RNA (TaKaRa Bio, Inc., Shiga, Japan) and 2 *μ*g total RNA was reverse transcribed with High‐Capacity cDNA Reverse Transcription Kit (Life Technologies, Inc., Tokyo, Japan). qRT‐PCR was performed using StepOnePlus^™^ Real‐Time PCR System (Applied Biosystems) and TaqMan probes, according to the manufacture's instruction.

### Western blotting analysis

ACC‐MESO‐4 and MSTO‐211H cells were seeded and incubated with afuresertib, as described above. Next, the cells were washed with ice‐cold PBS and were lysed in loading buffer (125 mmol/L Tris [pH 6.8], 4% SDS, 10% *β*‐mercaptoethanol, 20% glycerol, and 0.02% bromophenol blue). Western blotting analysis was performed as described previously 27. Relative protein levels were calculated after normalization with an internal control (*β*‐actin).

### Statistical analysis

At least three independent experiments and three replications per experiment were performed. Results are expressed as mean ± SE. Statistical significance between groups was determined using Student's *t*‐tests with SPSS 23.0 (SPSS Inc., Chicago, IL).

## Results

### Phosphorylated forms of Akt increase in MPM cells

**Figure 1:**
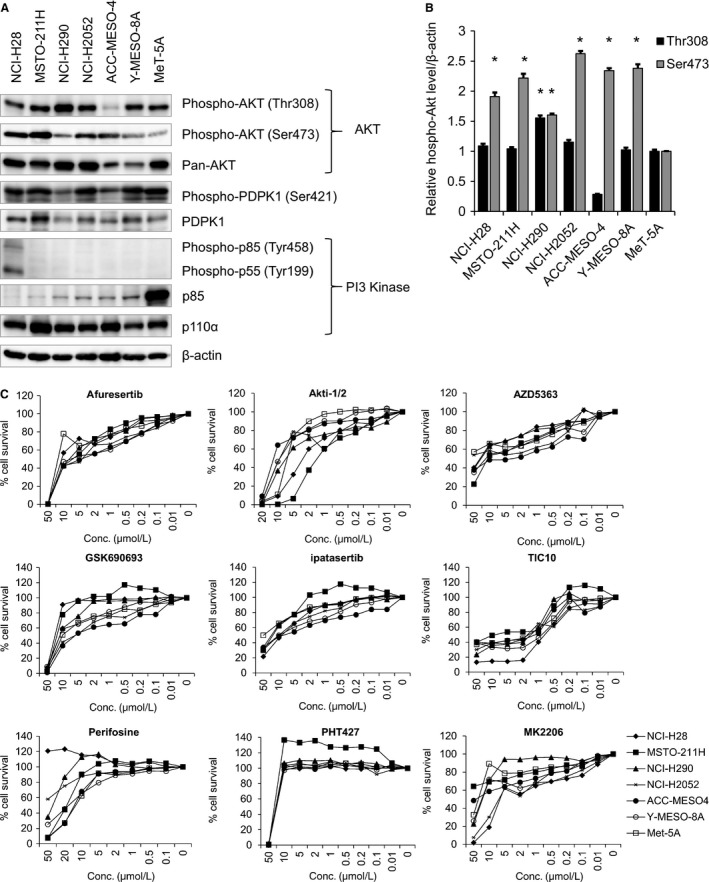
Phosphorylation level of Akt and effects of specific Akt inhibitors on the survival of MPM cells. (A) Six MPM cell lines, namely, ACC‐MESO‐4, MSTO‐211H, NCI‐H2052, NCI‐H28, NCI‐H290, and Y‐MESO‐8A, and a normal mesothelial cell line MeT‐5A were seeded in six‐well plates (cell density, 5 × 10^5^ cells/well). After 48 h, the cells were washed with PBS and were lysed in loading buffer. Cell lysates obtained were subjected to western blotting to detect phosphorylated Akt (Thr308 and Ser473), total Akt, phosphorylated PDPK1, PDPK1, phosphorylated p85/p55, p85, p110alpha, or *β*‐actin using specific antibodies listed in Table S1. (B) After normalization to *β*‐actin protein levels, the phosphorylation levels of Akt are expressed relative to the protein expression in the Met‐5A cells, which was arbitrarily defined as 1. The data are represented as the mean ± SE of three separate experiments. *indicates a statistically significant difference (*P *<* *0 .05). (C) The six MPM cell lines and the normal mesothelial cell line MeT‐5A were seeded in 96‐well plates (cell density, 2.5 × 10^3^ cell/well). On the following day, the cells were treated with the indicated concentrations (50 or 20, 10, 5, 2, 1, 0.5, 0.2, 0.1, and 0.01 *μ*mol/L) of Akt inhibitors (afuresertib, Akti‐1/2, AZD5563, GSK690693, ipatasertib, TIC10, perifosine, PHT427, and MK2206) for 72 h. Percentage survival of the six MPM cell lines was measured by performing the MTT assay. Data are expressed relative to the mean optic density (550 nm) of untreated cells, which was arbitrarily defined as 100%. Data are expressed as mean ± SE (*n* = 3).

To investigate Akt activation in MPM cells, we first examined the phosphorylation level of Akt (Thr308 and Ser473) in the six MPM cell lines ACC‐MESO‐4, MSTO‐211H, NCI‐H2052, NCI‐H28, NCI‐H290, and Y‐MESO‐8A and the normal mesothelial cell line MeT‐5A. Western blotting analysis showed that the phosphorylation of Akt (Thr308 and Ser473) increased in the MPM cell lines (Fig. 1A and B), indicating that Akt was frequently activated in MPM cells. We also examined the expression or phosphorylation level of upstream molecules in Akt signaling, including PDPK1, PI3K catalytic subunit p110*α*, and PI3K regulatory subunit p85. We found that PI3K/p110*α* expression increased in the MPM cell lines (Fig. 1A). In contrast, the expression and phosphorylation levels of PI3K/p85, which negatively regulates the catalytic activity of p110*α* 28, were negligible in the MPM cell lines except for NCI‐H28, whereas only total PI3K/p85 was detected in normal mesothelial cell line MeT‐5A (Fig. 1A). In addition, western blotting analysis clearly detected the phosphorylation level of PDPK1 in all the MPM cell lines and the normal mesothelial cell line (Fig. 1A). Together, these results suggest that PI3K/Akt signaling pathway is highly activated in MPM cells.

### Afuresertib, a potent Akt inhibitor, exhibits favorable tumor‐suppressive effects on MPM cells

**Figure 2:**
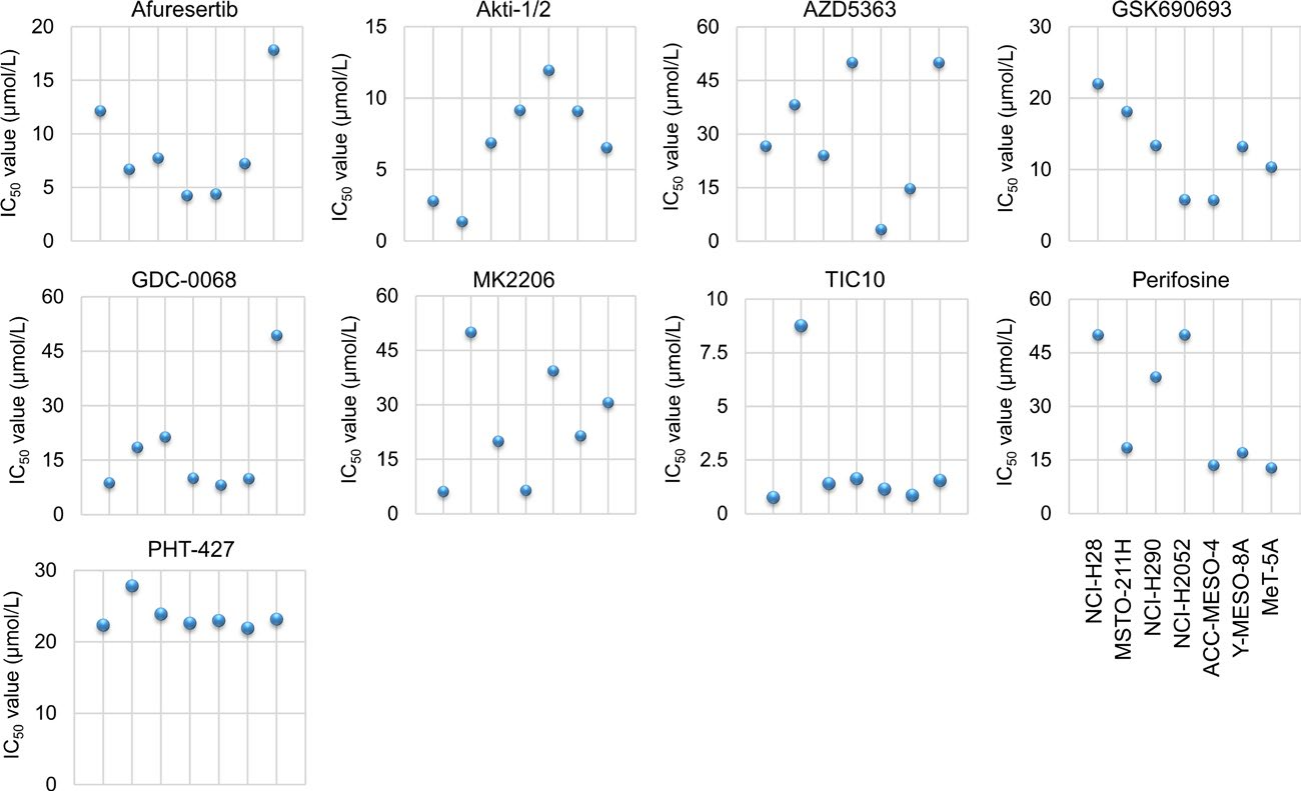
Comparison of antiproliferative efficacy of Akt inhibitors against MPM cells and MeT‐5A cells. The IC_50_ values (*μ*mol/L) of Akt inhibitors afuresertib, Akti‐1/2, AZD5563, GSK690693, ipatasertib, TIC10, perifosine, PHT427, and MK2206 against each cell line were determined by performing the MTT assay.

**Figure 3:**
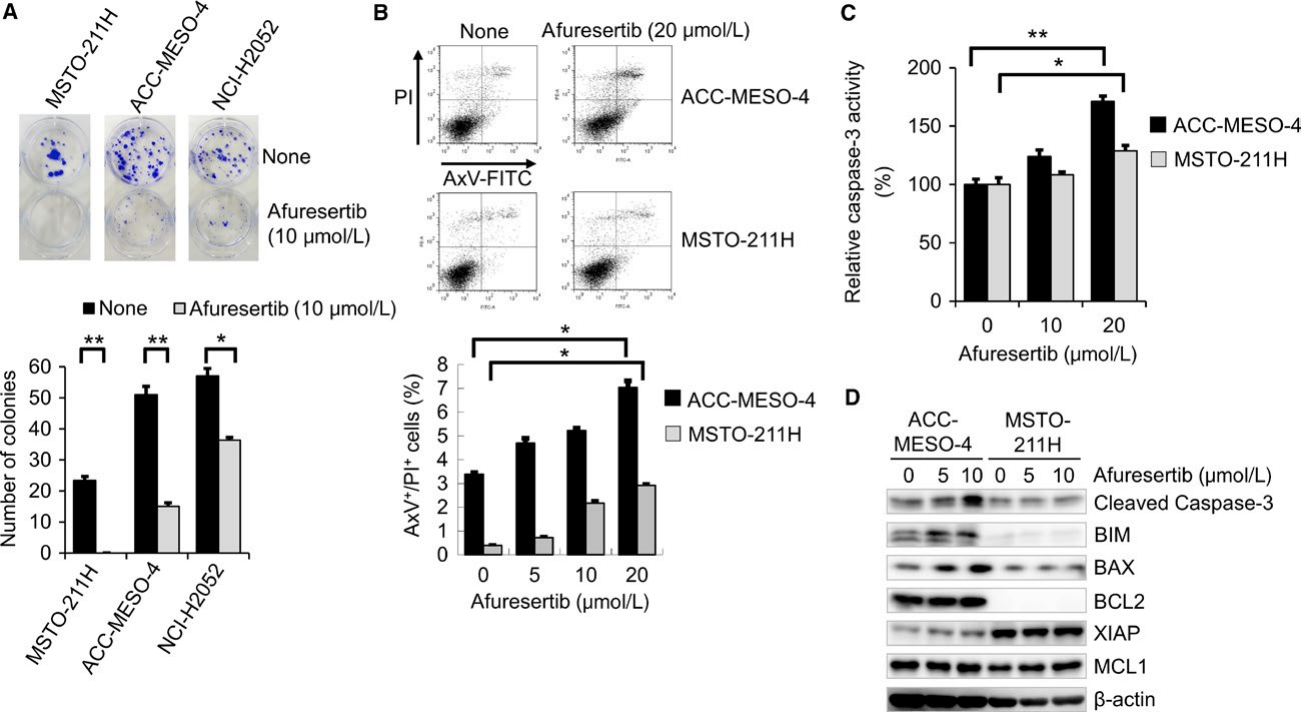
Effect of afuresertib on clonogenic activity and apoptosis of MPM cells. (A) Representative colony formation assay and results of the colony formation assay are shown. MSTO‐211H, ACC‐MESO‐4, and NCI‐H2052 cells (density, 200 cells/well) were seeded in a six‐well plate. On the following day, the cells were treated with 10 *μ*mol/L afuresertib for 14 days. Next, the cells were stained with crystal violet and were scanned. Bar graphs showing the number of stained colonies (*n* = 3). Data are expressed as mean ± SE (*n* = 3); ** or * indicates statistically significant difference (*P *<* *0.005 or *P *<* *0.05, respectively) compared with untreated cells. (B) Effect of afuresertib on apoptosis. ACC‐MESO‐4 and MSTO‐211H cells were treated with 20 *μ*mol/L afuresertib for 24 h. After incubation for 48 h, the cells were stained with AxV– FITC and PI. Percentages of AxV^+^/PI ^+^ cells are indicated; bar graphs showing the percentage of apoptotic (AxV^+^/PI ^+^) cells. Data are expressed as mean ± SE (*n* = 3). (C) Effect of afuresertib on caspase‐3 and caspase‐7 activities. ACC‐MESO‐4 and MSTO‐211H cells were treated with 0, 10, or 20 *μ*mol/L afuresertib for 24 h. Data are expressed relative to caspase‐3 and caspase‐7 activities in control cells (treated with 0 *μ*mol/L afuresertib), which was arbitrarily set at 100%; *indicates a statistically significant difference (*P *<* *0 .05). Data are expressed as mean ± SE (*n* = 3). (D) Effect of afuresertib on the expression of apoptosis‐related molecules. ACC‐MESO‐4 and MSTO‐211H cells were treated with 0, 5, or 10 *μ*mol/L afuresertib for 24 h. Next, the cells were washed with PBS and were lysed in loading buffer. Cell lysates obtained were subjected to western blotting analysis to detect cleaved caspase‐3, BIM, BAX, BCL2, XIAP, and MCL1 levels using specific antibodies listed in Table S1. *β*‐actin was used as internal control.

To investigate the involvement of Akt in MPM cell growth, we examined whether Akt inhibitors afuresertib, Akti‐1/2, AZD5363, GSK690693, ipatasertib (GDC‐0068), MK‐2206, perifosine, PHT‐427, and TIC10 affected the survival of the MPM cell lines and the normal mesothelial cell line. Target molecules, mechanisms of action, and structural features of the inhibitors are summarized in Table 1. Results of the MTT assay showed that treatment with all the Akt inhibitors suppressed cell viability in a dose‐dependent manner (Fig. 1C), suggesting that inhibition of Akt activity suppressed MPM cell growth. To determine the tumor specificity of the nine Akt inhibitors, we examined the IC_50_ values of these inhibitors against MPM cells. The IC_50_ values of the Akt inhibitors are summarized in Table 2 and Figure 3. Notably, we found that the IC_50_ values of both afuresertib and GDC‐0068 against all the MPM cell lines were lower than those against the normal mesothelial cell line MeT‐5A (Fig. 2), suggesting that these inhibitors exerted tumor‐specific antiproliferative effect on MPM cells. Akt is recruited to the plasma membrane by phosphatidylinositol (3,4,5) trisphosphates (PIP_3_), which is produced by PI3K. Akt is then phosphorylated at T308 and S473 by phosphoinositide‐dependent kinase‐1 (PDPK1) and mTORC2, respectively 29. Therefore, we examined the effect of PI3K inhibitor PF04691502 and PDPK1 inhibitor OSU‐03012 on the survival of MPM cells by performing the MTT assay. We found that both PF04691502 and OSU‐03012 suppressed MPM cell survival in a dose‐dependent manner (Fig. S1A). Moreover, we found that the IC_50_ values of PF04691502 but not OSU‐03012 against all the MPM cell lines were lower than those against the normal mesothelial cell line MeT‐5A (Fig. S1B).

**Table 1:** Summary of AKT inhibitors used in this study

Inhibitors	Target molecules	Mechanism of action	Structural feature
Afuresertib	AKT1, AKT2, AKT3	catalytic, ATP‐competitive inhibitor	thiophenecarboxamide derivatives
Akti‐1/2	AKT1, AKT2	allosteric inhibitor	2,3‐diphenylquinoxaline core
AZD5363	AKT, PKA, mTOR	catalytic, ATP‐competitive inhibitor	heterocyclic rings
GSK690693	AKT	catalytic, ATP‐competitive inhibitor	Aminofurazan
Ipatasertib	AKT	catalytic, ATP‐competitive inhibitor	heterocyclic rings
MK‐2206	AKT	allosteric inhibitor	2,3‑diphenylquinoxaline analogue
OSU‐03012 (AR‐12)	PDK‐1	catalytic, ATP‐competitive inhibitor	celecoxib (COX‐2 inhibitor)‐derived small‐molecule
Perifosine	AKT	allosteric inhibitor	alkylphospholipid
PF‐04691502	PI3K	catalytic, ATP‐competitive inhibitor	4‐methylpyridopyrimidinone derivatives
PHT‐427	AKT	allosteric inhibitor	sulfonamide derivative
TIC10	AKT, ERK	unknown	imidazo[1,2‐a]pyrido[4,3‐d]pyrimidine derivative

**Table 2:** IC_50_ values (µM) of AKT inhibitors against each cell line

Cell lines	NCI‐H28	MSTO‐211H	NCI‐H290	NCI‐H2052	ACC‐MESO‐4	Y‐MESO‐8A	Met‐5A
Race^a^	Caucasian	Caucasian	Unknown	Caucasian	Japanese	Japanese	Unknown
Histological subtypes^a^	Epithelial	Biphasic	ND	Epithelial	Epithelial	Biphasic	Normal mesothelium^b^
AKT inhibitors							
Afuresertib	12.1	6.7	7.7	4.2	4.4	7.2	17.8
Akti‐1/2	2.8	1.4	6.9	9.2	12.0	9.10	6.5
AZD5363	26.6	38.2	24.0	>50	3.3	14.7	>50
GSK690693	22.0	18.1	13.4	5.8	5.7	13.2	10.4
Ipatasertib	8.7	18.5	21.4	10.0	8.1	9.9	49.4
MK‐2206	6.1	>50	20.0	6.5	39.3	21.4	30.6
OSU‐03012	3.1	0.89	2.6	1.3	0.45	0.43	1.2
Perifosine	>50	18.4	38.2	>50	13.5	17.0	12.8
PF‐04691502	0.06	0.16	0.30	0.21	0.30	0.25	0.83
PHT‐427	22.3	27.8	23.9	22.6	23.0	21.9	23.2
TIC 10	0.76	8.8	1.4	1.6	1.1	0.86	1.6

a The race and histological subtypes of cell lines used in this study are indicated by referring to Hakiri et al. 36.

b Met‐5A cell line was established by introducing SV40 early region DNA to normal human mesothelial cells 37.

### Afuresertib suppresses clonogenic activity and induces apoptosis

Because the IC_50_ values for afuresertib against the MPM cell lines were approximately 50% lower than those of GDC‐0068, we used afuresertib for performing subsequent experiments. We examined the tumor‐suppressive effect of afuresertib on ACC‐MESO‐4, MSTO‐211H, and NCI‐H2052 cells. Afuresertib significantly inhibited colony formation by ACC‐MESO‐4, MSTO‐211H, and NCI‐H2052 cells (*P *<* *0.005 for ACC‐MESO‐4 and MSTO‐211H and *P *<* *0.05 for NCI‐H2052; Fig. 3A). The Akt inhibitor perifosine suppresses MPM cell growth 30. Therefore, we examined the effect of afuresertib and perifosine on the clonogenicity. Afuresertib but not perifosine significantly suppressed colony formation by ACC‐MESO‐4 and MSTO‐211H cells (Figs. S2 A and B). In addition, perifosine but not afuresertib significantly decreased the number of colonies formed by normal mesothelial MeT‐5A cells (Figs. S2 A and B). These results strongly suggest that afuresertib exerts favorable tumor‐suppressive effects against MPM cells. We further examined the effect of afuresertib on apoptosis in ACC‐MESO‐4 and MSTO‐211H cells. The percentages of AxV^+^/PI^+^ cells significantly increased among both ACC‐MESO‐4 and MSTO‐211H cells after 48 h of afuresertib treatment (Fig. 3B). In addition, afuresertib significantly increased caspase‐3 and caspase‐7 activities in ACC‐MESO‐4 cells (Fig. 3C). Moreover, afuresertib slightly increased caspase‐3 and caspase‐7 activities in MSTO‐211H cells (Fig. 3C). Similarly, results of western blotting analysis showed that afuresertib increased the levels of proapoptotic molecules, including BIM and BAX, and cleaved caspase‐3 in ACC‐MESO‐4 cells (Fig. 3D). These results suggest that afuresertib‐induced inhibition of MPM cell proliferation is associated with apoptosis induction.

### Effect of afuresertib on cell cycle status and Akt‐related molecule expression in MPM cells

**Figure 4:**
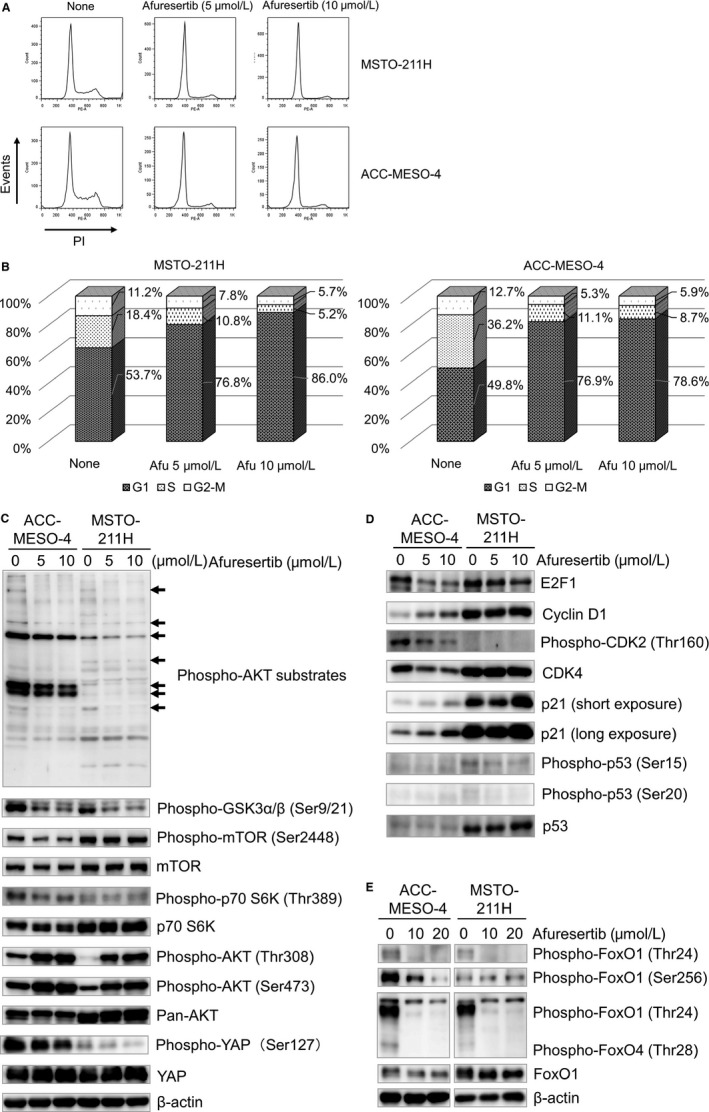
Effect of afuresertib on cell cycle and phosphorylation levels of Akt substrates and Akt‐related molecules. (A and B) ACC‐MESO‐4 and MSTO‐211H cells (density, 1 × 10^5^ cells/well) were seeded in six‐well plate. After incubation for 48 h, the cells were treated with 0, 5, 10 *μ*mol/L afuresertib for 24 h. Next, the cells were detached, fixed, and stained with 100 *μ*g/mL PI after treatment with 1 mg/mL RNase. Populations of cells in the different phases of the cell cycle were measured using FACSCanto II and FlowJo software. A representative FACS histogram (A) and bar graphs showing the ratio of cells in the different phases of the cell cycle (B). (C‐E) Effect of afuresertib on the phosphorylation levels of Akt substrates (C), cell cycle‐related molecules (D), and FOXO proteins. ACC‐MESO‐4 and MSTO‐211H cells were treated as described in Fig. 3D. Cell lysates obtained were subjected to western blotting analysis with specific antibodies listed in Table S1. *β*‐actin was used as internal control.

Inhibition of Akt activity was reported to distribute cell cycle properties 31. Therefore, we examined the effect of afuresertib on cell cycle status by performing PI‐staining‐based FACS analysis. Afuresertib dramatically increased the population of cells in the G_0_/G_1_ phase and decreased the population of cells in the S and G_2_‐M phases of the cell cycle (Fig. 4A). Results of the cell confluence proliferation assay showed that afuresertib suppressed cell confluency in a dose‐dependent manner (Fig. S3), suggesting that afuresertib arrested cell cycle in the G_1_ phase. We next investigated the effect of afuresertib on the expression and/or phosphorylation levels of downstream molecules, including Akt substrates and cell cycle regulators. Phosphorylation levels of Akt substrates GSK3*β* (Ser9/21), mTOR (Ser2448), and p70 (Thr389) decreased after afuresertib treatment (Fig. 4C). Interestingly, phosphorylation level of YAP, a transcriptional factor in the Hippo signaling pathway, decreased after afuresertib treatment (Fig. 4C). Moreover, phosphorylation levels of Akt (Thr308 and Ser473) increased after afuresertib treatment (Fig. 4C). In addition, afuresertib decreased the levels of E2F1 and CDK4 and phosphorylation level of CDK2 and increased the level of p21^WAF1/CIP1^, a cell cycle regulator in the G_1_ phase (Fig. 4D). p53 is a well‐known inducer of p21^WAF1/CIP1^. In this study, we did not observe any increase in the phosphorylation levels of p53 (Ser15 and Ser20) (Fig. 4D). FOXO1, an Akt substrate, potentiates p21 expression after undergoing dephosphorylation. Therefore, we examined changes in the phosphorylation level of FOXO1. As expected, we observed that the phosphorylation level of FOXO1 (Thr24 and Ser256) decreased after afuresertib treatment (Fig. 4E). The effect of afuresertib on the migration of MPM cells was determined by performing the scratching assay with ACC‐MESO‐4 and MSTO‐211H cells. We found that afuresertib (5 *μ*mol/L) clearly suppressed the migration of MPM cells (Figure S4). These results strongly suggest that afuresertib inhibits the growth of MPM cell and decreases the phosphorylation of several direct and/or indirect Akt substrates.

### Afuresertib and cisplatin treatment cooperatively suppress cell survival and induce apoptosis

**Figure 5:**
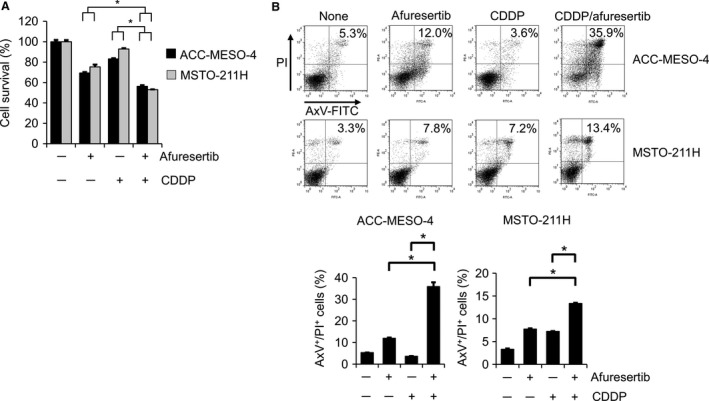
Effect of afuresertib and cisplatin combination treatment on cell viability and apoptosis. (A) ACC‐MESO‐4 and MSTO‐211H cells were seeded in a 96‐well plate (cell density, 2.5 × 10^3^ cells/well). On the following day, the cells were treated with afuresertib (20 *μ*mol/L) and/or cisplatin (20 *μ*mol/L for ACC‐MESO‐4 cells and 10 *μ*mol/L for MSTO‐211H cells) for 72 h. MTT analysis to determine the rate of cell growth was performed as described in Fig 1B. Data are presented relative to the mean optical density (550 nm) of untreated cells, which was arbitrarily defined as 100%. Data are expressed as means ± SE (*n* = 3). (B) After incubation for 48 h, the cells were stained with AxV‐FITC and PI. Representative dot plots are shown above; horizontal line, PI (FL‐2); Vertical line, AxV‐FITC (FL‐1).

Cisplatin (CDDP) is one of the most frequently used anticancer drugs for MPM treatment 32. Therefore, we examined whether afuresertib affected the cytotoxic effect of CDDP. Results of the MTT assay showed that afuresertib and CDDP combination treatment significantly suppressed the viability of both ACC‐MESO‐4 and MSTO‐211H cells compared with afuresertib or CDDP monotherapy (Fig. 5A). This novel finding prompted us to examine the effect of afuresertib and CDDP combination treatment on apoptosis induction. As expected, the combination treatment significantly induced apoptosis (Fig. 5B), indicating that afuresertib‐induced Akt inhibition enhanced the cytotoxic effect of CDDP on MPM cells. We also examined whether afuresertib affected the cytotoxic effect of pemetrexed on MPM cells. Results of the MTT assay showed that pemetrexed treatment decreased the survival of MSTO‐211H cells but not of ACC‐MESO‐4 cells (Fig. S5A). Therefore, we examined the effect of afuresertib and pemetrexed combination treatment on apoptosis induction in MSTO‐211H cells. Result of flow cytometry analysis showed that afuresertib significantly enhanced pemetrexed‐induced apoptosis in ACC‐MESO‐4 cells (Fig. S5B).

### Afuresertib downregulates the expression of a subset of genes with oncogenic signatures

**Figure 6:**
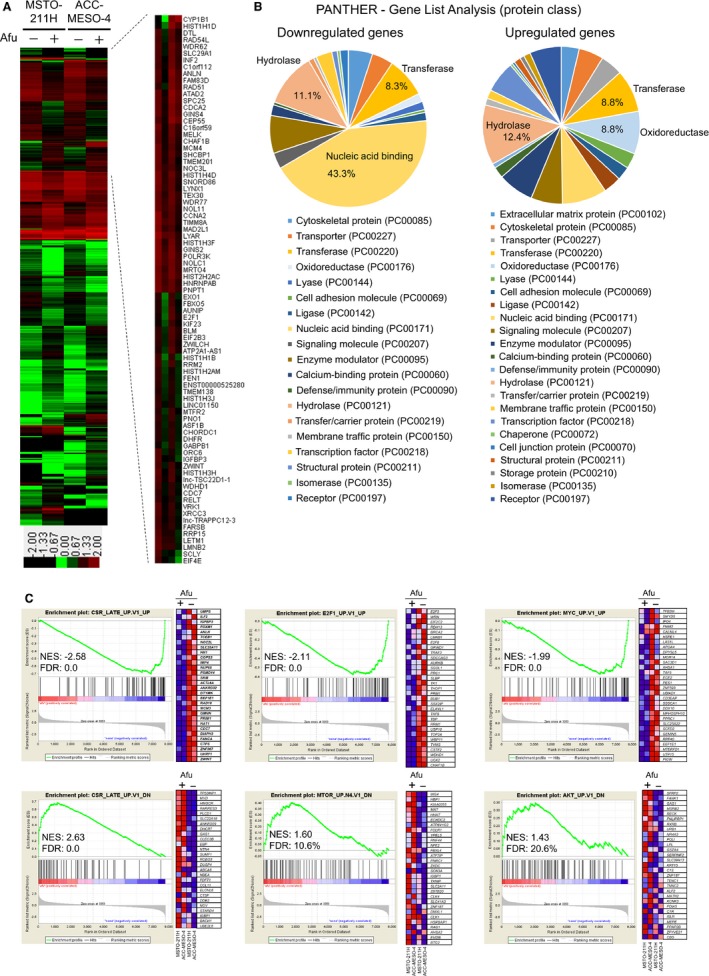
Gene expression analysis. MSTO‐211H and ACC‐MESO‐4 cells were incubated with or without afuresertib (10 *μ*mol/L) for 24 h. Next, total RNA was extracted and cDNA microanalysis was performed using SurePrint G3 Human 8 × 60K V3 format (Agilent). (A) Heatmap of the upregulated genes (262 genes; fold change, >2.0) and downregulated genes (219 genes, fold change <0.5) after afuresertib treatment. The heatmap was constructed using normalized values of each sample with Treeview software. A heatmap showing downregulated genes, with the corresponding gene name at the right side, after afuresertib treatment. (B) Gene ontology analyses using the Panther classification system. The upregulated and/or downregulated genes were classified using PANTHER ‐ Gene List Analysis (www.pantherdb.org/). (C) GSEA was conducted using GSEA v2.2.4 software and Molecular Signatures Database (Broad Institute). All raw data were formatted and applied to oncogenic signatures (C6). Representative GSEA enrichment plots and corresponding heatmap images of the indicated gene sets in 10 *μ*mol/L afuresertib‐treated and untreated cells, respectively, are shown. Genes contributing to enrichment are shown in rows, and the sample is shown in one column on the heatmap. Expression level is represented as a gradient from high (red) to low (blue). FDR, false discovery rate; NES, normalized enrichment score.

To clarify the effect of afuresertib on gene expression in MPM cells, we conducted a comprehensive cDNA microarray analysis. Heatmap analysis showed similar gene expression patterns of afuresertib‐treated MSTO‐211H and ACC‐MESO‐4 cells (Fig. 6A). Fold change analysis showed that afuresertib upregulated the expression of 262 genes by >2.0 fold and downregulates the expression of 219 genes by <0.5 fold compared with that in untreated cells (Tables S2 and Table S3). PANTHER classification analysis showed that 43.3% of the downregulated genes encoded nucleic acid‐binding protein (Fig. 6B). Interestingly, we found that 76.7% of the downregulated genes encoding nucleic acid‐binding protein are further classified into a protein class of DNA binding protein (data not shown), suggesting that afuresertib may affect DNA‐protein interaction in MPM cells. Therefore, we performed gene set enrichment analysis (GSEA) to investigate whether the expression of a specific set of oncogenesis‐associated genes was significantly different between afuresertib‐treated and untreated MPM cells. Result of GSEA of genes with oncogenic signatures showed significant inactivation of Akt signaling‐associated genes (*SFRP2*,* FANK1*, and *GAS1*), serum‐responsive genes (e.g., *ZWINT*,* UHRF1*, and *CDC7*), E2F1‐induced genes (e.g. *CHAF1B*,* UCK2*, and *TOP2A*), MYC‐induced genes (e.g., *PIGW*,* UTP15*, and *SLC25A22*), and mTOR‐related genes (e.g., *ING4*,* HBP1*, and *KIAA0355*) (Fig. 6C). We also found that afuresertib significantly decreased the expression of cell cycle‐dependent kinase 1 gene (*CDK‐1*), *CDK‐2*,* E2F1*, and insulin‐like growth factor‐binding protein 3 gene (*IGFBP3*) but not of *GAPDH* in MPM cells (Fig. S6). GSEA using Kyoto Encyclopedia of Genes and Genomes database also showed significant inactivation of genes associated with spliceosome‐, DNA replication‐, and cell cycle‐related signaling (Fig. S7). These results strongly suggest that afuresertib suppresses MPM cell proliferation by modulating the expression genes associated with oncogenic signaling. Collectively, our results suggest that afuresertib exerts promising tumor‐suppressive effect on MPM cells.

## Discussion

MPM is associated with an increasing incidence rate in industrially advanced countries. Despite advancements in medical techniques for treating MPM, it is still a highly incurable disease. Therefore, new molecular targeting therapies are warranted to effectively treat patients with MPM. In this study, we examined the effects of a panel of Akt inhibitors on the survivals of MPM cell lines and an immortalized normal mesothelial cell line and found that afuresertib exerts favorable tumor‐suppressive effects. We also found that afuresertib decreased the phosphorylation and/or total protein levels of Akt substrates and/or cell cycle‐related molecules. Moreover, we found that afuresertib significantly downregulates the expression of genes with oncogenic signatures.

Akt inhibitors are classified into two groups and are currently being developing for clinical use 33. One group of Akt inhibitors includes catalytic, ATP‐competitive inhibitor (such as afuresertib) that preferentially target active kinases. Another group of AKT inhibitors includes allosteric inhibitor (such as perifosine) that binds to the N‐terminal pleckstrin homology domain of Akt. These inhibitors induce conformational changes in Akt that prevents its membrane localization and subsequent activation 34. Recently, Pinton et al. reported that the allosteric Akt inhibitor perifosine suppressed MPM cell growth by inhibiting EGFR/MET‐Akt signaling axis 30. In this study, we found that the catalytic, ATP‐competitive Akt inhibitor afuresertib suppressed the growth and clonogenic activity of MPM cells in a dose‐dependent manner. Similar results were obtained with another catalytic, ATP‐competitive Akt inhibitor ipatasertib. Analysis of IC_50_ values indicated that the dose of afuresertib required for suppressing MPM cell growth was lower than that of perifosine. Our observation that the average of IC_50_ value of afuresertib against MPM cell lines was lower than that against MeT‐5A strongly suggests that afuresertib preferentially affects MPM cell growth compared with perifosine.

PI3K is a well‐known upstream molecule of Akt and plays an important role in activating Akt 6. In this study, we found that the catalytic, ATP‐competitive PI3K inhibitor PF04691502 dose‐dependently suppressed MPM cell survival, with IC_50_ values similar to those of afuresertib. This result indicates that PI3K‐Akt signaling performs a tumorigenic role in MPM cells.

ATP‐competitive Akt inhibitors, including A‐443654 and GSK GSK690693, induce paradoxical hyperphosphorylation of Akt at its two regulatory sites Thr308 and Ser473 35. In this study, we found that afuresertib enhanced the phosphorylation of Akt at Thr308 and Ser473, which is consistent with that observed using other catalytic, ATP‐competitive Akt inhibitors, including GDC‐0068 33 Results of gene expression profiling performed in this study showed that afuresertib significantly suppresses oncogenic gene expression related to serum response, E2F1, MYC, mTOR, as well as Akt. This result suggests that afuresertib‐induced hyperphosphorylation does not activate of Akt‐mediated signaling. We also found that afuresertib increased the expression of gene sets associated with inositol phosphate and/or amino acid metabolisms, including branched chain amino acids (BCAAs) and tryptophan metabolism, suggesting that these metabolic pathways are alternatively used to supply cellular energy after Akt inhibition in MPM cells. Therefore, it would be interesting to examine whether inhibition of inositol phosphate and/or BCAA metabolic pathways enhances afuresertib‐induced suppression in MPM cells.

Akt activation induces resistance to chemotherapy 19, 20. Our finding that afuresertib enhanced CDDP‐induced tumor suppression strongly suggests that afuresertib prolongs the chemosensitivity of MPM cells and/or overcomes chemoresistance to CDDP in patients with MPM. Pinton et al. reported that the combination of perifosine with CDDP but not with pemetrexed exerted synergistic tumor‐suppressive effect on MPM cells 30.

In conclusion, this study is the first to show that afuresertib decreased the survival of MPM cells by inhibiting Akt. Our results suggest that the catalytic, ATP‐competitive Akt inhibitor afuresertib preferentially inhibits MPM cell growth compared with allosteric inhibitors such as perifosine. It would be interesting to investigate molecular mechanism through which afuresertib exerts tumor‐specific cytotoxicity. Our novel finding that afuresertib enhanced CDDP‐induced cytotoxic effects provides an attractive option of using afuresertib and cisplatin combination therapy for treating MPM. Because our data were mainly obtained from in vitro studies, it is necessary to assess the anticancer activity of afuresertib in vivo, for example, by establishing xenograft models. Moreover, further studies are required to develop novel molecular targeting therapies for MPM treatment.

## Conflict of Interest

The authors declare that they do not have any conflicts of interest related to this study.

## Supporting information

Figure S1. The effect of specific PI3K (PF‐04691502) or PDPK1 (OSU‐03012) inhibitor on cell survival in MPM cell lines.
Figure S2. The comparison of antitumor effect of afuresertib and perifosine in MPM cell lines (MSTO‐211H and ACC‐MESO‐4) and normal mesothelium cell line (MeT‐5A).
Figure S3. Cell confluence proliferation assay.
Figure S4. Scratching assay. ACC‐MESO‐4 (A) and MSTO‐211H (B) cells were seeded in 24‐well plates (1 × 105cells/well) and incubated for 24 h at 37°C.
Figure S5. Combinatorial effect of afuresertib with pemetrexed treatment on cell viability and apoptosis.
Figure S6. The results of cDNA microarray analysis.
Figure S7. GSEA analysis with kyotoencyclopedia of genes and genomes (KEGG) gene sets.

Table S1. Antibodies used in this study

Table S2. Downregulated genes after afuresertib treatment in MPM cell lines.

Table S3. Upregulated genes after afuresertib treatment in MPM cell lines.
