# Supplementary material for: Novel ATP‐competitive Akt inhibitor afuresertib suppresses the proliferation of malignant pleural mesothelioma cells
Source: Cancer Med. 2017 Sep 27;6(11):2646–59. doi: 10.1002/cam4.1179 (PMC5673922; doi:10.1002/cam4.1179)
Supplement: Supplementary file 1 — Figure S1. The effect of specific PI3K (PF‐04691502) or PDPK1 (OSU‐03012) inhibitor on cell survival in MPM cell lines. Figure S2. The comparison of antitumor effect of afuresertib and perifosine in MPM cell lines (MSTO‐211H and ACC‐MESO‐4) and normal mesothelium cell line (MeT‐5A). Figure S3. Cell confluence proliferation assay. Figure S4. Scratching assay. ACC‐MESO‐4 (A) and MSTO‐211H (B) cells were seeded in 24‐well plates (1 × 10^5^cells/well) and incubated for 24 h at 37°C. Figure S5. Combinatorial effect of afuresertib with pemetrexed treatment on cell viability and apoptosis. Figure S6. The results of cDNA microarray analysis. Figure S7. GSEA analysis with kyotoencyclopedia of genes and genomes (KEGG) gene sets. [file CAM4-6-2646-s001.pdf]

Supplementary Figure S1

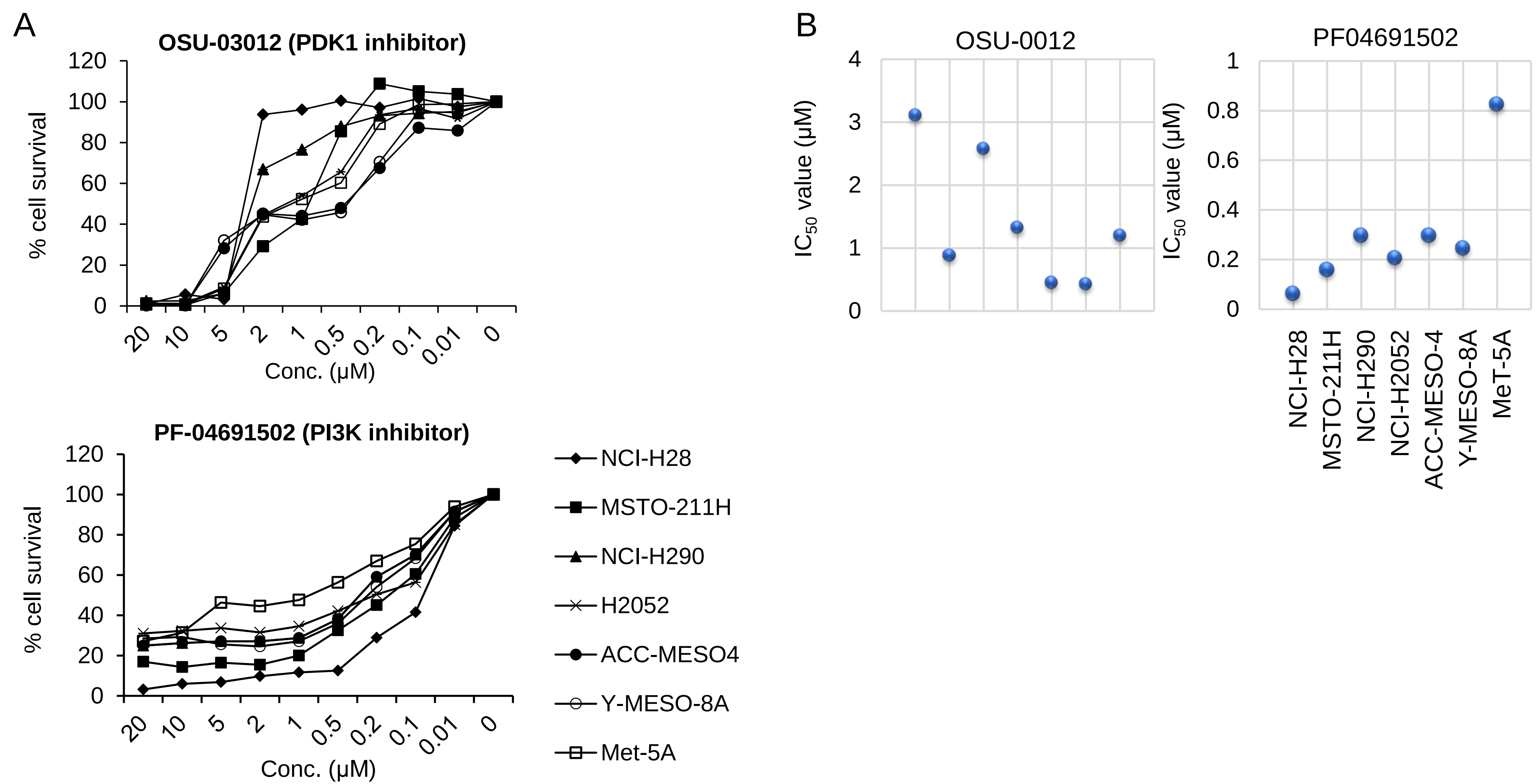

sFIg. S1. **The effect of specific PI3K (PF-04691502) or PDPK1 (OSU-03012) inhibitor on cell survival in MPM cell lines.** (A) Six MPM cell lines and MeT-5A cells were seeded in 96-well plates (2.5 × 103 cell per well). On the following day, the cells were treated with the indicated concentrations (50 or 20, 10, 5, 2, 1, 0.5, 0.2, 0.1, and 0.01 μM) of Akt inhibitors (afuresertib, Akti-1/2, AZD5563, GSK690693, ipatasertib, TIC10, perifosine, PHT427, and MK2206) for 72 h. The percentage of cell survival of 6 MPM cell lines were measured by MTT assay. Data are expressed relative to the mean optic density (550 nm) found in the untreated cells, which was arbitrarily defined as 100%. Data are expressed as the mean ± SE (n = 3). (B) The IC_{50} values (μM) of the inhibitors were determined for each cell line according to the results of MTT assay.

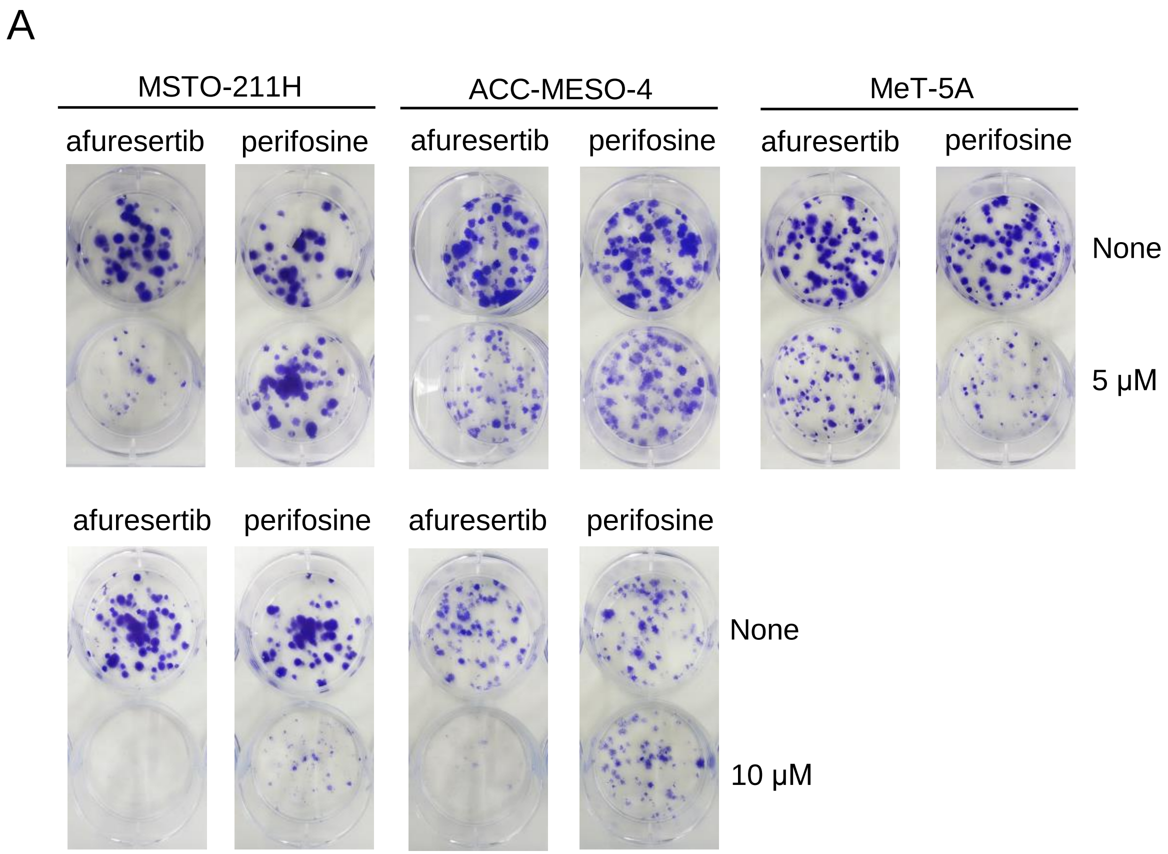

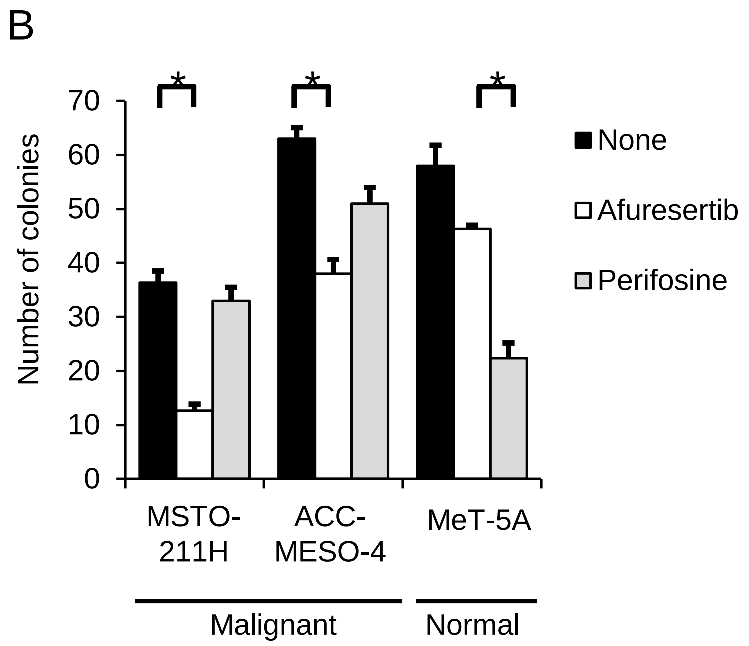

sFig. S2. **The comparison of anti-tumor effect of afuresertib and perifosine in MPM cell lines (MSTO-211H and ACC-MESO-4) and normal mesothelium cell line (MeT-5A).** (A-B) Representative colony formation assay (A) and results of the colony formation assay (B) are shown. MSTO-211H, ACC-MESO-4, and MeT-5A cells (200 cells/well) were seeded in a 6-well plate. On the following day, the cells were treated with afuresertib (10 μM) for 14 days. After incubation for 14 days, the cells were stained with crystal violet and scanned. Bar graphs showing the number of stained colonies are presented (n = 3). Data are expressed as the mean ± SE (n = 3). Asterisk (*) indicates statistically significant difference of $P < 0.05$, compared to no treatment, respectively.

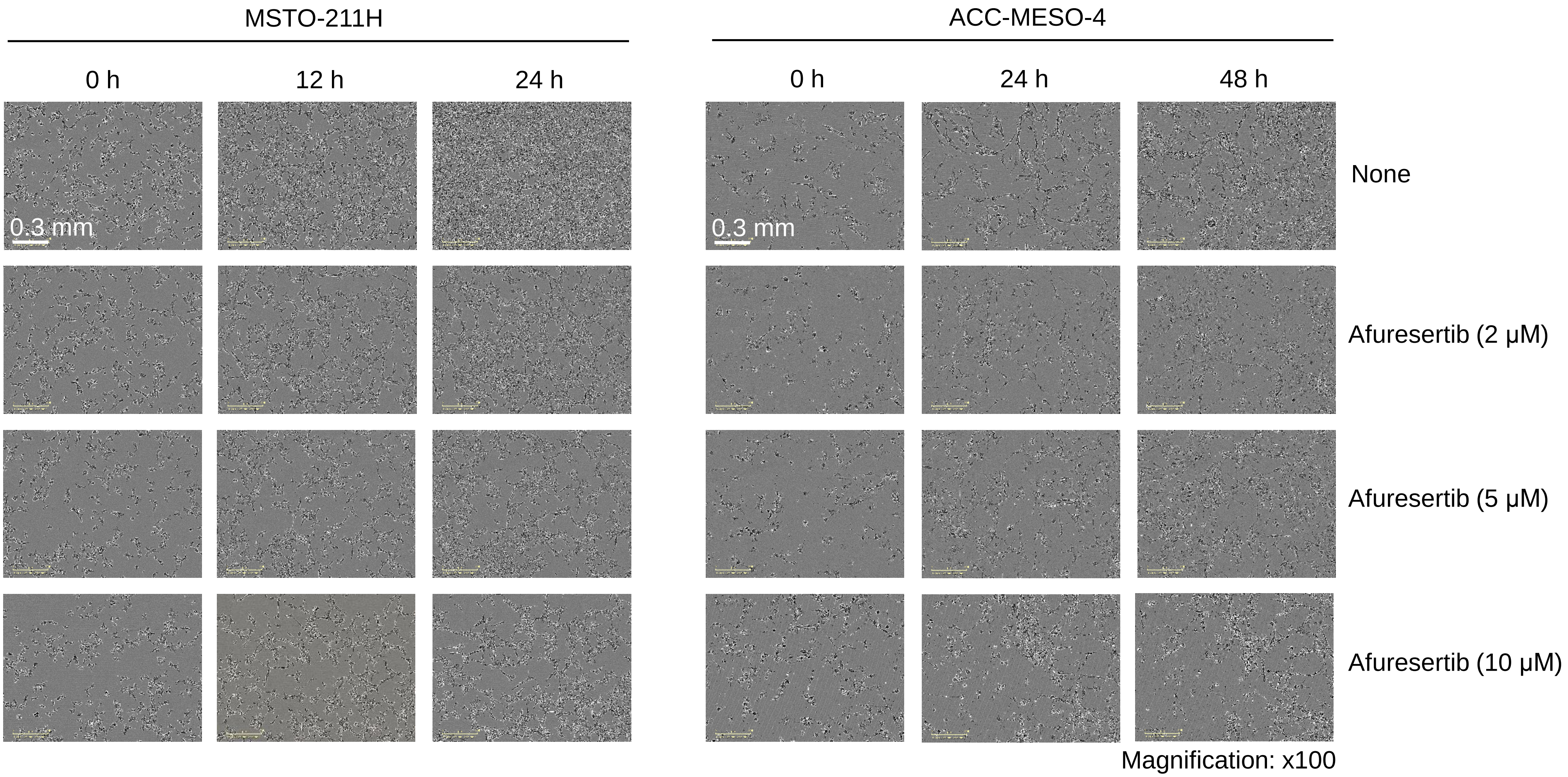

sFIg. S3. **Cell confluence proliferation assay**. ACC-MESO-4 and MSTO-211H cells were seeded in 12-well plates (1 × 10^4 cells/well) and incubated for 24 h at 37°C. Then, the cells were incubated with the indicated concentrations (0, 2, 5, 10 μM) of afuresertib. During treatment, the cell growth were monitored by recording phase images using the IncuCyte ZOOM® live cell imaging system (magnification: x100).

Supplementary Figure S4

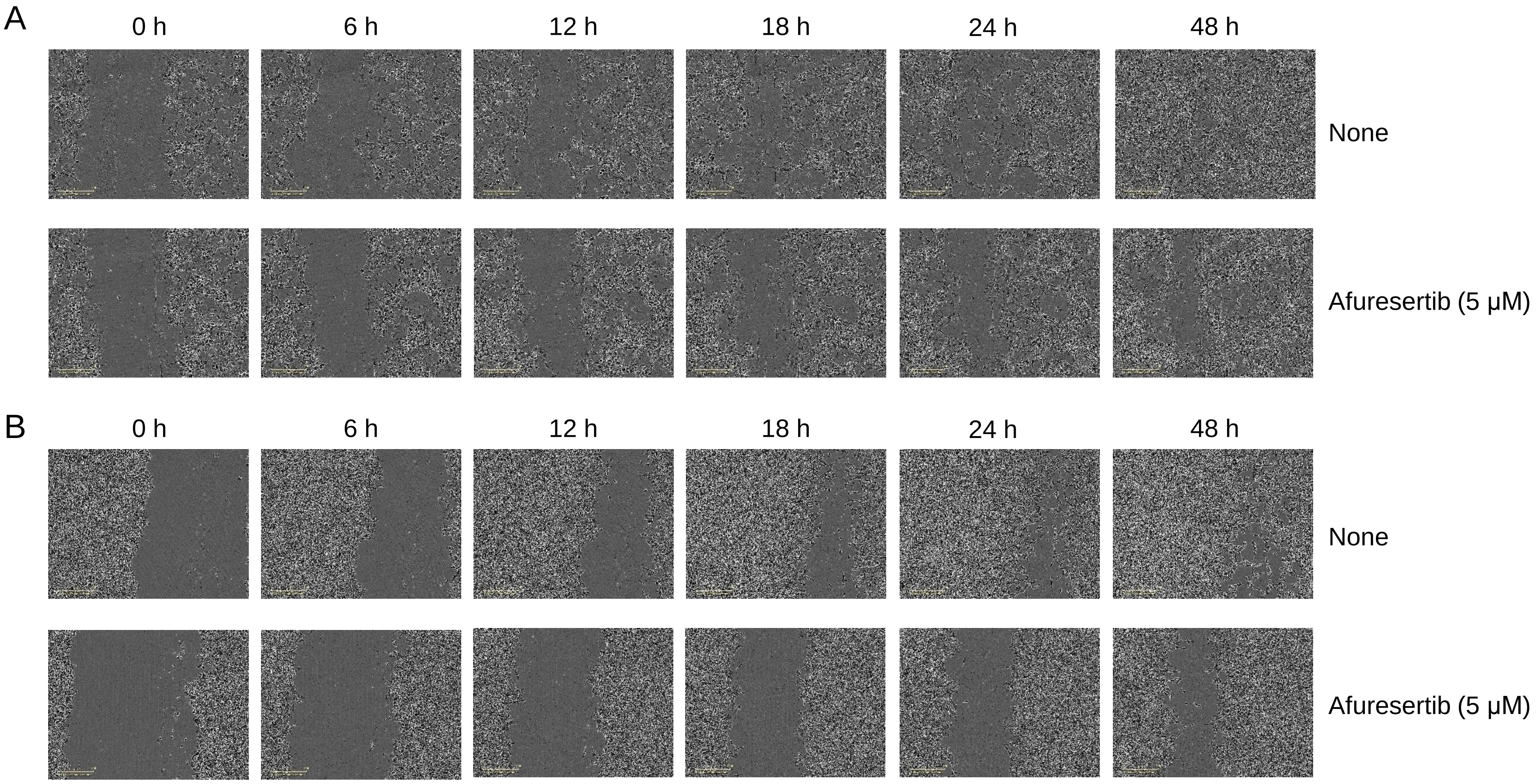

sFIg. S4. **Scratching assay**. ACC-MESO-4 (A) and MSTO-211H (B) cells were seeded in 24-well plates (1×10^5 cells/well) and incubated for 24 h at 37°C. When the cell density reached 80-90% confluence, the cells were gently scratched with a new 1 ml pipette tip across the center of the well. After scratching, cell culture medium was replaced to fresh medium containing indicated concentration of afuresertib. Visual validation of treatments was performed using IncuCyte™ ZOOM System (Essen BioScience, Inc., Ann Arbor, MI, USA).

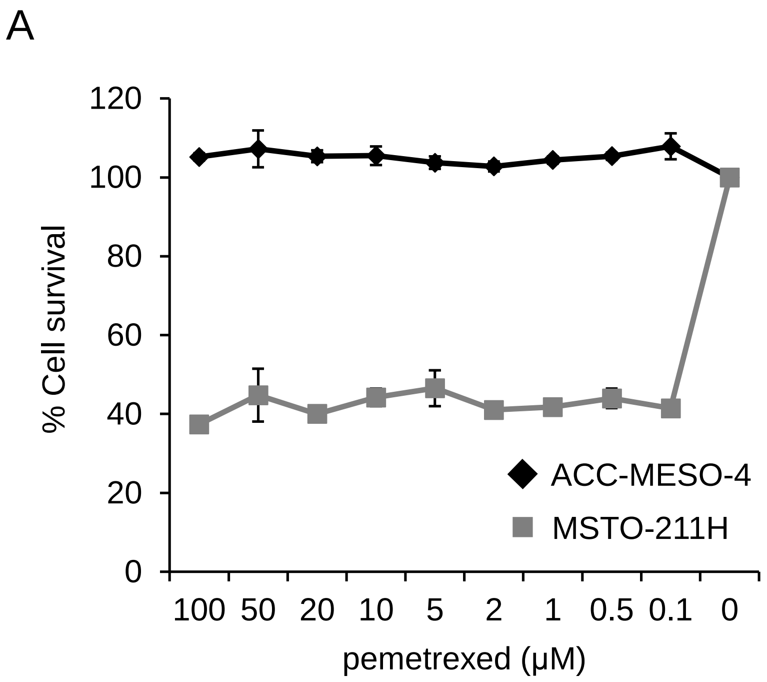

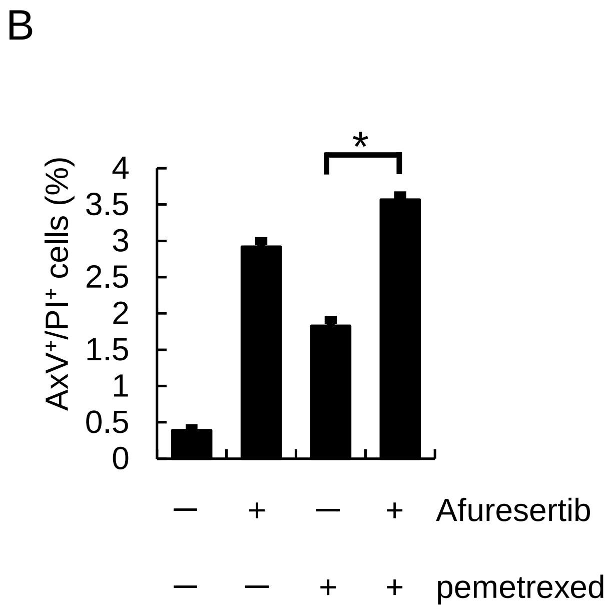

sFig. S5. **Combinatorial effect of afuresertib with pemetrexed treatment on cell viability and apoptosis.** (**A**) ACC-MESO-4 and MSTO-211H cells were seeded in a 96-well plate (2.5×10^3 cells/well). On the following day, the cells were treated with the indicated concentration of pemetrexed (100, 50, 20, 10, 5, 2, 1, 0.5, 0.1, and 0 μM) for 72 h. MTT analysis of the growth rate was performed as described in Fig 1B. Data are presented relative to the mean optical density (550 nm) in the untreated cells, which was arbitrarily defined as 100%. Data are expressed as means ± SE (n=3). (B) After incubation for 48 h, the cells were stained with AxV-FITC and PI. Bar graphs showing the percentage of apoptosis (AxV^+/PI^+ cells) are presented. Data are represented as the mean ± SE (n=3). *$P < 0.05$, significant difference.

Supplementary Figure S6

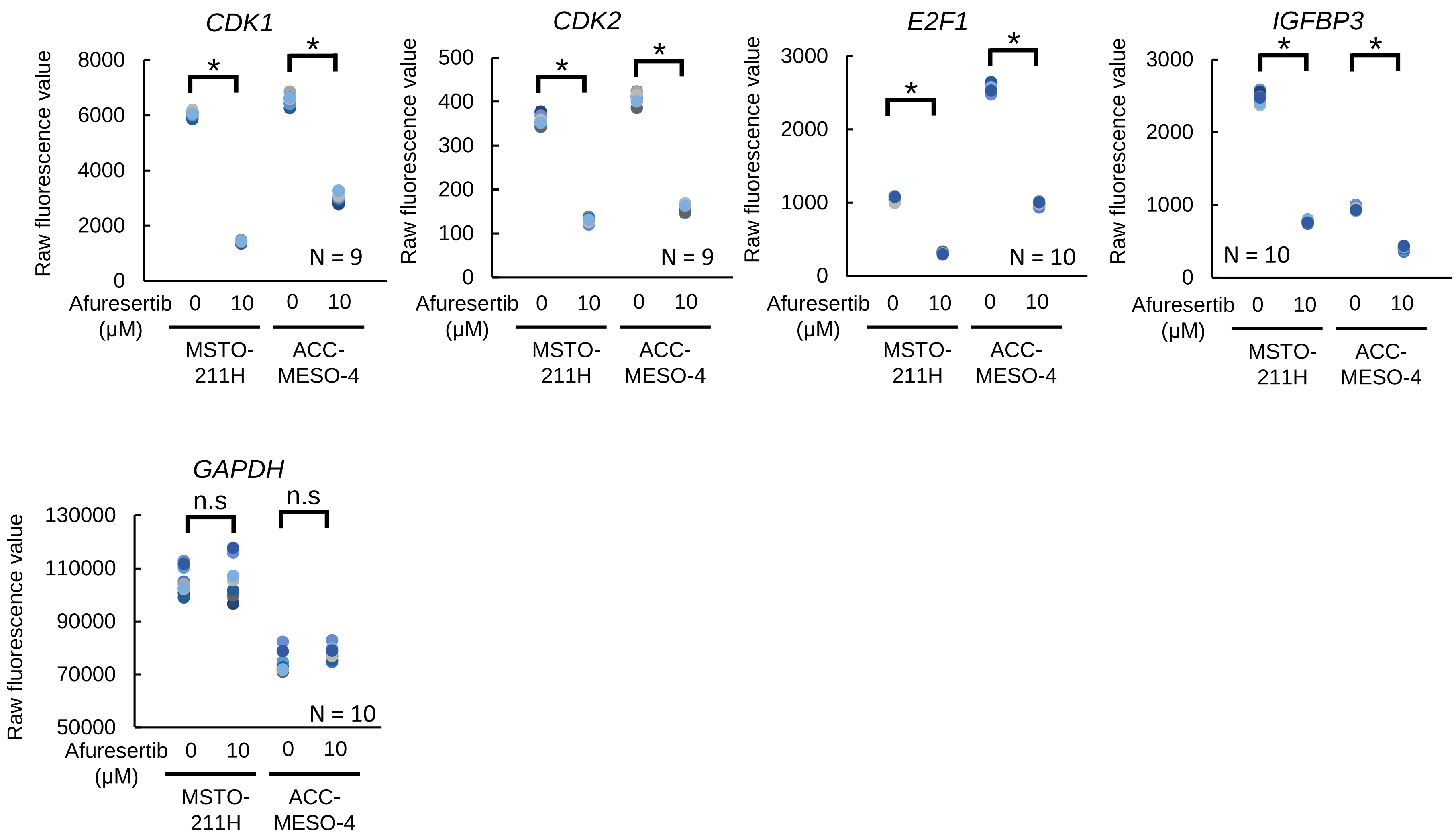

sFig. S6. **The results of cDNA microarray analysis**. The graphs show the differential gene expression between afuresertib treated cells and untreated cells. Raw fluorescence values obtained by scanning were utilized for comparison of gene expression. Representative genes related to cell cycle (*CDK1* and *CDK2*) and Akt signaling pathway (*E2F1* and *IGFBP3*) are shown. *$P < 0.05$, significant difference.

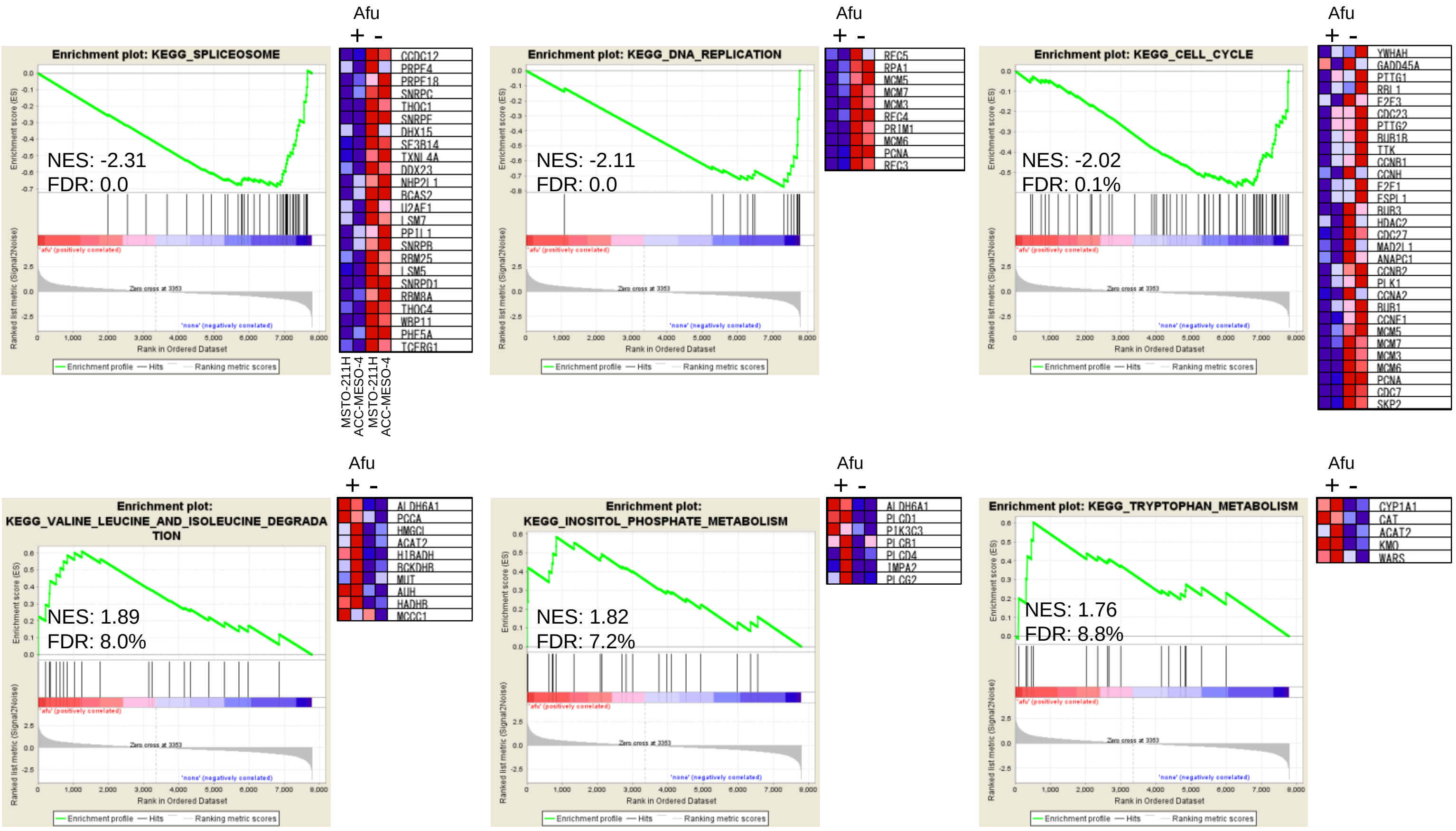

ClassA: afuresertib treatment (10 μM)

ClassB: no treatment

sFig. S7. **GSEA analysis with kyoto encyclopedia of genes and genomes (KEGG) gene sets**. GSEA analysis were conducted using GSEA v2.2.4 software and the Molecular Signatures Database (Broad Institute). All the raw data were formatted and applied to KEGG gene sets (C2).
